# EFFECTS OF DISCRIMINATION ACROSS THE LIFE COURSE: A CASE STUDY OF NARRATIVE METHOD ON TAIWANESE MOVIE BANANA PARADISE

**DOI:** 10.1093/geroni/igae098.3838

**Published:** 2024-12-31

**Authors:** Sheng-Chin HSU

**Affiliations:** Ming Chuan University, Taipei, Taipei, Taiwan (Republic of China)

## Abstract

The effects of discrimination across the life course are one of the products from the cumulations of diverse individuals and groups. This paper aims to present the results of effects of discrimination across the life course on Taiwan movie “Banana Paradise” through a narrative theory. “Banana Paradise” expressed the changes of main character in his life course, and showed diversity of his identity and discrimination via these changes during 1949 to 1989. The main objective is to offer an alternative to understand what effects of discrimination are constructed by the shifting across the life course and at the same time to show how effects of discrimination were embodied by interactions with others across one’s life course.

